# A Schisandra-Derived Compound Schizandronic Acid Inhibits Entry of Pan-HCV Genotypes into Human Hepatocytes

**DOI:** 10.1038/srep27268

**Published:** 2016-06-02

**Authors:** Xi-Jing Qian, Xiao-Lian Zhang, Ping Zhao, Yong-Sheng Jin, Hai-Sheng Chen, Qing-Qiang Xu, Hao Ren, Shi-Ying Zhu, Hai-Lin Tang, Yong-Zhe Zhu, Zhong-Tian Qi

**Affiliations:** 1 Department of Microbiology, Shanghai Key Laboratory of Medical Biodefense, Second Military Medical University, Shanghai 200433, China; 2 State Key Laboratory of Virology, Medical Research Institute of Wuhan University and Department of Immunology and Hubei Province Key Laboratory of Allergy and Immunology, Wuhan University School of Medicine, 185 Donghu Road, Wuhan 430071, Hubei Province, China; 3 Department of Organic Chemistry, College of Pharmacy, Second Military Medical University, Shanghai 200433, China; 4 Department of Phytochemistry, College of Pharmacy, Second Military Medical University, Shanghai 200433, China

## Abstract

Despite recent progress in the development of hepatitis C virus (HCV) inhibitors, cost-effective antiviral drugs, especially among the patients receiving liver transplantations, are still awaited. Schisandra is a traditional medicinal herb used to treat a range of liver disorders including hepatitis for thousands of years in China. To isolate the bioactive compounds of schisandra for the treatment of HCV infection, we screened a schisandra-extracts library and identified a tetracyclic triterpenoid, schizandronic acid (SZA), as a novel HCV entry inhibitor. Our findings suggested that SZA potently inhibited pan-HCV genotype entry into hepatoma cells and primary human hepatocytes without interfering virus binding on cell surface or internalization. However, virion-cell fusion process was impaired in the presence of SZA, along with the increased host membrane fluidity. We also found that SZA inhibited the spread of HCV to the neighboring cells, and combinations of SZA with interferon or telaprevir resulted in additive synergistic effect against HCV. Additionally, SZA diminished the establishment of HCV infection *in vivo*. The SZA target is different from conventional direct-acting antiviral agents, therefore, SZA is a potential therapeutic compound for the development of effective HCV entry inhibitors, especially for patients who need to prevent HCV reinfection during the course of liver transplantations.

Hepatitis C virus (HCV) is a major cause of chronic liver diseases, with an estimated prevalence of 3% of the world’s population^1^. Currently, no vaccine is available for the prevention of HCV infection, and the treatment of hepatitis patients with pegylated interferon-alpha (IFN-α) and ribavirin (RB) has limited efficacy especially in genotype 1 and 4 infections^2^. Very recently, an addition to the therapy of novel direct-acting antivirals (DAAs) such as protease inhibitors telaprevir, boceprevir or simeprevir, and RNA polymerase inhibitor sofosbuvir have shown to increase the sustained virological response (SVR) rate in HCV patients^3^,^4^,^5^. In 2014, the use of ledipasvir/sofosbuvir (Harvoni) and the interferon-free regimen including a combination regimen of ombitasvir/paritaprevir/ritonavir and dasabuvir tablets (Viekira Pak) was approved by the Food and Drug Administration (FDA) for treating genotype 1 patients^6^,^7^,^8^. However, these therapeutic options of individual antiviral drugs are likely to result in resistance development and also carry significant side effects^9^. Thus, novel combinations of drugs targeting different stages of the HCV life cycle, especially virus entry, may provide a promising approach against HCV drug resistance development and infection relapse.

The entry of HCV into hepatocytes is a highly coordinated process involving multiple factors including viral envelope glycoproteins and host cell machinery. Glycosaminoglycans (GAGs) and low-density lipoprotein receptor (LDL-R) have been proposed to concentrate HCV particles on the surface of hepatocytes^10^,^11^. Furthermore, four specific entry factors including scavenger receptor class B type I (SRB1), tetraspanin CD81, tight junction proteins claudin-1 (CLDN1) and occludin (OCLN) are sequentially involved after the initial phase of virus binding to the surface of hepatocytes^12^. Virions are later internalized via clathrin-mediated endocytosis and fused with the host membrane in the late endosome stage^13^. In addition, host factors Niemann Pick C1 Like1 (NPC1L1), transferrin receptor 1 (TfR1), epidermal growth factor receptor (EGFR) and ephrin receptor A2 (EphA2) have also been implicated in HCV entry into hepatocytes^14^,^15^,^16^.

The fruit of *Schisandra sphenanthera Rehd. et Wils.* (schisandra) and its extracts have been used as traditional medicine in East Asia to treat liver disorders such as hepatitis. They have also been reported to possess a wide spectrum of biological and pharmacological properties, including antiviral, anti-inflammatory and anti-oxidative properties, without any associated toxicities^17^. Schisandra has been used as an adjuvant drug in Chinese clinics for decades. In a phase I clinical trial, a combination therapy of multiple anti-oxidants including schisandra extracts reduced one order of magnitude of HCV RNA level in 25% of patients with chronic HCV infection in the study^18^ . These promising results prompted us to identify the anti-HCV compounds from the fruit of schisandra. In this study, schizandronic acid (SZA), a tetracyclic triterpene, extracted from the fruit of *Schisandra sphenanthera Rehd. et Wils.*, inhibited pan-genotypic HCV entry into human hepatocytes by interfering with virion-cell membrane fusion.

## Results

### Schisandra screening for potential HCV entry inhibitor

**Figure 1:**
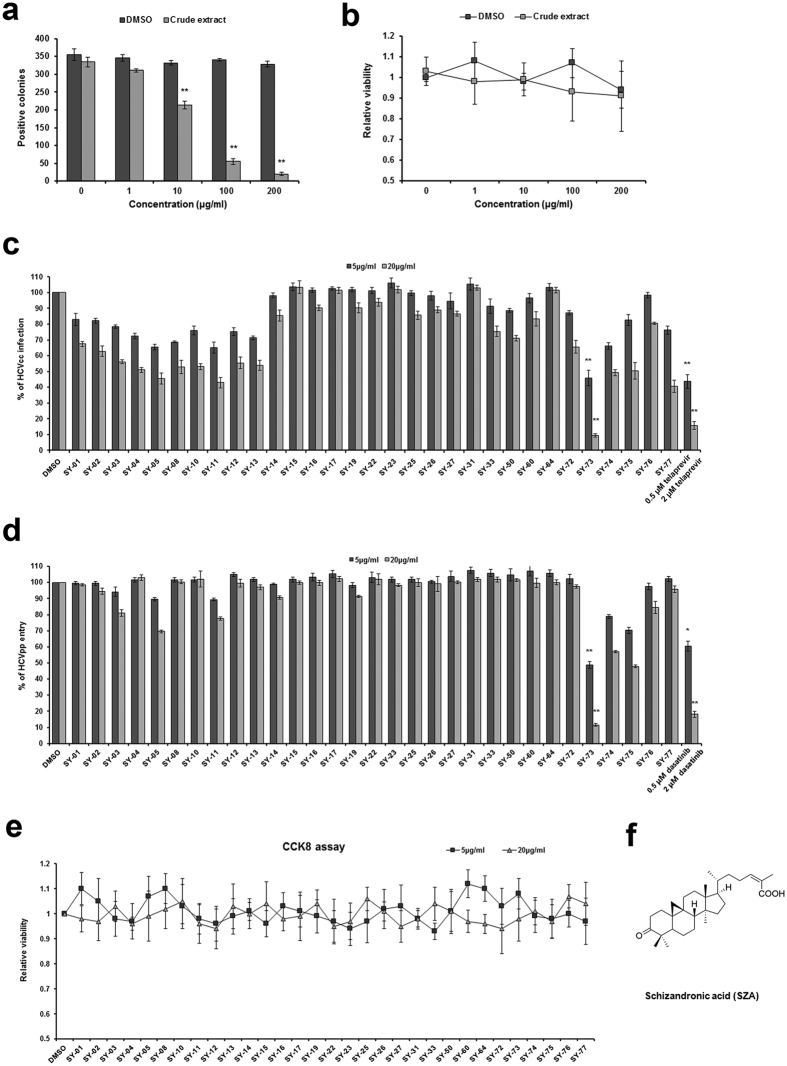
Selection of anti-HCV entry inhibitor. (**a**) Huh7 cells were infected with HCVcc of 2a JFH-1 (multiplicity of infection MOI = 1) with increasing concentrations of crude extract of schisandra for 4 h. At 48 h post-infection, the infectivity was analyzed by IF. (**b**) Cell viability of crude extract of schisandra. (**c,d**) Anti-HCV activity of schisandra-derived compounds (5/20 μg/ml) using HCVcc of 2a JFH-1 (MOI = 1) or HCVpp of 1a H77 for 4 h. 0.5/2 μM telaprevir or dasatinib was introduced as a positive control for HCVcc infection or HCVpp entry. At 48 h post-infection, HCVcc infection was determined by IF. At 72 h post-incubation, HCVpp entry was determined by flow cytometry. Results are plotted as % of infection/entry compared to DMSO treated group in parallel. *p < 0.05, **p < 0.01 compared to DMSO control group. (**e**) Cell viability of schisandra-derived compounds (5/20 μg/ml) using CCK8. Results are plotted as relative viability compared to DMSO treated group. Data shown as mean ± SD of three independent experiments. (**f**) Chemical structure of SZA.

The crude extract of *Schisandra sphenanthera Rehd. et Wils* was analyzed for its antiviral activity during HCV infection in human hepatoma Huh7 cells. As shown in Fig. 1a, the extract exhibited an inhibitory effect at a concentration of 10 μg/ml and above, with low cytotoxicity (Fig. 1b). To identify the bioactive compounds with antiviral activity, natural compounds were isolated from the schisandra fruit extract (Supplementary Table), and anti-HCV activity as well as cytotoxicity were evaluated in Huh7 cells (Fig. 1c–e). Huh7 cells were incubated with JFH-1 HCVcc of 2a strain or H77 HCVpp of 1a strain in the presence of the fruit extracts for 4 h. Among these compounds, the tetracyclic triterpene SY-73, also known as SZA (Fig. 1f), inhibited both HCVcc infection and HCVpp entry by approximately 90% at a concentration of 20 μg/ml with low cytotoxicity (Fig. 1c–e), a potent entry inhibitor to be selected for further study.

### SZA inhibits entry of major HCV genotypes into primary hepatocytes

**Figure 2:**
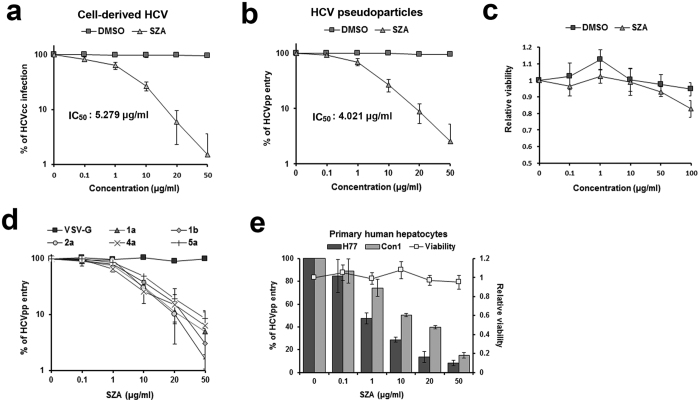
Antiviral activity of SZA is dose-dependent and pan-genotypic, and its inhibitory effect is active in PHHs. (**a,b**) Anti-HCV activity using Huh7 cells infected with HCVcc of JFH-1 strain (MOI = 1) or HCVpp of H77 strain together with the indicated concentrations of SZA or DMSO for 4 h. Half maximal inhibitory concentration (IC_50_) of SZA for HCVcc infection or HCVpp entry is listed in the figures. Results are shown as % of HCVcc infection or HCVpp entry compared to DMSO treated group in parallel. (**c**) Cell viability of SZA using Huh7 cells treated with the indicated concentrations of SZA for 24 h by CCK8. (**d**) Antiviral effect of SZA on HCVpp of different genotypes. Huh7 cells were treated with the indicated concentrations of SZA together with HCVpp of different genotypes or VSV-G for 4 h. At 72 h post-incubation, HCVpp entry was detected by flow cytometry. Results are plotted as % of HCVpp entry compared to untreated group. (**e**) Anti-HCV activity and viability of SZA in PHHs. The PHHs were treated with the indicated concentrations of SZA together with HCVpp of 1a H77 strain or 1b Con1 strain for 4 h. At 72 h post-incubation, the entry rate was measured by flow cytometry. Results are plotted as % of HCVpp entry and relative viability compared to untreated group. Data shown as mean ± SD of three independent experiments.

The structure and purity of SZA (SY-73) was confirmed by mass spectrometry (MS), nuclear magnetic resonance (NMR) analysis and high performance liquid chromatography (HPLC) (Supplementary Fig. 1). Our results indicated that SZA inhibited HCVcc infection and HCVpp entry into Huh7 cells in a dose-dependent manner with the IC_50_ of 5.279 μg/ml and 4.021 μg/ml respectively without obvious cytotoxicity (Fig. 2a–c). However, SZA did not show inhibitory effect on Japanese encephalitis virus (JEV) infection (Supplementary Fig. 2), suggesting that its antiviral activity was unique to HCV entry.

To test the genotype-specific inhibitory effect of SZA on HCV entry, HCVpp harboring E1E2 proteins of different HCV strains were produced. As shown in Fig. 2d, SZA inhibited the infectivity of HCVpp from different genotypes and subtypes, indicating that HCV entry inhibition occurred independent of HCV genotype. In the case of the pseudoparticles of vesicular stomatitis virus (VSV) that harbors a different class of fusion proteins from HCV, SZA showed no obvious inhibitory effect on it.

The primary human hepatocytes (PHHs) closely resemble the hepatocytes functioning as the main reservoir of HCV within the infected host. As the PHHs do not support robust replication of HCV genomes *in vitro*, they were incubated in the presence of SZA with HCVpp of 1a strain H77 or 1b strain Con1 carrying a green fluorescent protein reporter gene. The entry of HCVpp into the PHHs was strongly inhibited by SZA in a dose-dependent manner (Fig. 2e) without obvious cytotoxicity. Thus, SZA was also able to impede HCV entry into its natural target cells.

### SZA does not affect HCV replication, assembly and release

**Figure 3:**
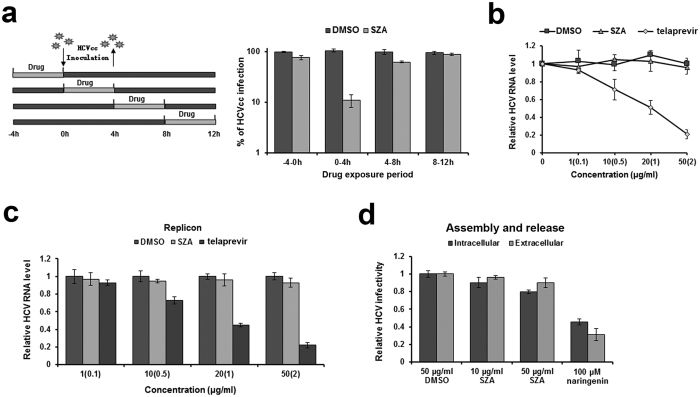
SZA has no inhibitory effect on viral replication, assembly and release. (**a**) Huh7 cells were infected with HCVcc of JFH-1 (MOI = 1) and treated by 20 μg/ml of SZA for 4 h during indicated time periods as shown in the left panel. At 48 h post-infection, infected cells were performed IF. Results are plotted as % of HCVcc infection compared to DMSO treated group in parallel. (**b**) Antiviral activity of SZA on replication. JFH-1 RNA was electroporated into Huh7 cells. At 4 h after electroporation, the cells were treated with the indicated concentrations of SZA for 4 h at 37 ^o^C. Concentrations of telaprevir are shown in brackets of the figure. Results are shown as relative HCV RNA level compared to untreated group. (**c**) BB7 replicon cells were incubated with the indicated concentrations of SZA or telaprevir for 4 h. Viral RNA levels were determined by qPCR 48 h after treatment. Concentrations of telaprevir are in brackets of the figure. Results are plotted as relative HCV RNA level compared to DMSO treated group. (**d**) Antiviral activity of SZA on assembly and release. Huh7 cells were electroporated with JFH-1 RNA of HCV, followed by 4 h treatment of the indicated concentrations of SZA. At 48 h after electroporation, cells were subjected to three cycles of freeze and thaw to test the intracellular viral infectivity; supernatants of the electroporated cells were collected for the detection of extracellular viral infectivity. 100 μM naringenin was introduced as a positive control. Results are shown as relative HCV infectivity compared to DMSO treated group. Data shown as mean ± SD of three independent experiments.

The above screening assay suggested that SZA might act during the early stage of the HCV life cycle. Moreover, it was also observed that SZA inhibited HCV infection most potently when added to the cells together with the virus, but showed low inhibitory effect when introduced 4 h before or after the infection (Fig. 3a). Therefore, it is necessary to check whether SZA may have additional roles in the other steps of the viral life cycle. To evaluate the effect of SZA on HCV RNA replication, we transfected Huh7 cells with subgenomic JFH-1 viral RNA. Increasing doses of SZA were added 4 h later, and replication efficiency was assessed 48 h after the transfection using RT-qPCR. HCV RNA level in replication deficient strain JFH-1 (GND) transfected cells was used as a baseline of the transfected RNA, and was barely detectable in qPCR assay (data not shown). Huh7 cells were incubated with increasing doses of telaprevir as control. No significant changes in HCV RNA levels were observed after SZA treatment (Fig. 3b). We also used a HCV BB7 replicon cell line to test the influence of SZA on viral replication and did not observe any significant changes (Fig. 3c). To determine whether SZA affects viral assembly or release, we transfected Huh7 cells with JFH-1 RNA, and quantified the intracellular and extracellular viral infectivity by reinfection of Huh7 cells 72 h after transfection using both the cell supernatants and lysate taken by repeated freeze and thaw assay. As shown in Fig. 3d, SZA did not reduce intracellular or extracellular viral infectivity up to a dose of 50 μg/ml, while naringenin, an assembly inhibitor, diminished both intracellular and extracellular viral infectivity. These data indicate that SZA does not impede HCV RNA replication, assembly and release.

### SZA inhibits HCV entry into the host cells at post-binding stage

**Figure 4:**
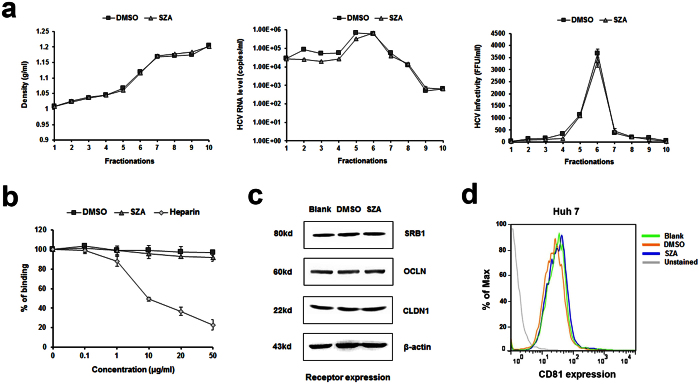
SZA does not affect virus integrity, binding or entry factors expression. (**a**) Concentrated HCVcc of JFH-1 strain was incubated with SZA (20 μg/ml) or DMSO at 37 °C for 2 h before the compound was removed by filters. Iodixanol gradient ultracentrifugation was then performed. Each of the ten gradient fractions was weighed to calculate density (left panel). HCV RNA levels were determined by RT-qPCR (middle panel) and viral infectivity by reinfection of naïve Huh7 cells (right panel). (**b**) The effect of SZA on HCV binding. Huh7 cells were incubated with HCVcc at 4 ^o^C for 2 h to facilitate virus binding. Indicated concentrations of either SZA or heparin was added. The quantity of bound viral particles was determined by RT-qPCR. Results are plotted as % of binding compared to untreated group. Data shown as mean ± SD of three independent experiments in the above figures. (**c**) The effect of SZA on the expression levels of cellular entry factors of HCV. Huh7 cells were incubated with SZA (50 μg/ml) for 4 h, and the expression levels of SRB1, CLDN1 and OCLN were determined by western blotting, (**d**) and CD81 by flow cytometry 24 h post-incubation.

To assess whether SZA acts through perturbation of lipoprotein association or virion integrity, we incubated HCVcc in the presence of SZA for 4 h before iodixanol step gradient centrifugation. The virus was then loaded on a continuous iodixanol gradient (10–40%) for 16 h ultracentrifugation at 146,000 g at 4 ^o^C. The fractions were collected, weighed and assayed for HCV RNA level and viral infectivity. No major changes in density distribution or viral RNA level or infectivity were observed (Fig. 4a). These results suggest that SZA utilized an alternative mechanism to block HCV entry by acting on the host cell machinery.

Subsequently, we detected the effect of SZA on virus binding. Huh7 cells were inoculated with purified HCVcc at 4 ^o^C in the presence of SZA, and the quantity of bound virions was determined by qPCR. Heparin was used as a positive control for virus binding inhibition. As expected, heparin strongly reduced HCV attachment to the cell surface, while in the presence of SZA, no effect on virus binding was observed (Fig. 4b). These results indicate that SZA plays no role in the impairment of HCV binding to the host cell surface.

To analyze the mechanism by which SZA inhibits HCV entry, we assessed the expression of known essential HCV entry factors CD81, SRB1, CLDN1, and OCLN. Huh7 cells were treated with SZA at 50 μg/ml for 4 h. Then, CD81, SRB1, CLDN1 and OCLN expression were assessed by western blotting or flow cytometry 24 h post-incubation. The expression levels of all four entry factors were found unaltered, indicating that SZA does not act by down-regulating these entry factors (Fig. 4c,d).

**Figure 5:**
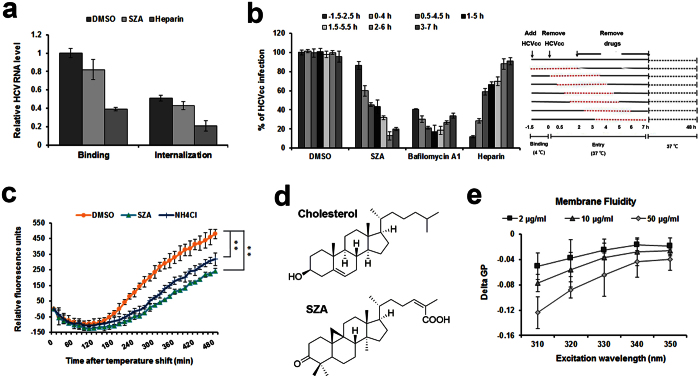
SZA impairs virion-cell membrane fusion during HCV post-binding process. (**a**) The effect of SZA on viral internalization. Huh7 cells were incubated with HCVcc at 4 °C for 90 min for virus binding in the presence of SZA (20 μg/ml). Half of the cells were lysed to determine the bound viral particles, and the other cells were placed in a 37 ^o^C incubator for 30 min to allow the internalization of viral particles followed by the trypsinization for 1 h on ice to remove the non-internalized virions. Quantities of the bound and the internalized viral particles were determined by RT-qPCR. Heparin was applied as a positive control for virus binding. Results are plotted as relative HCV RNA level compared to DMSO treated group during binding. (**b**) Huh7 cells were incubated with HCVcc of JFH-1 at 4 ^o^C for 1.5 h to allow virus binding. The plate was then placed in a 37 ^o^C incubator for synchronized virus entry. SZA (20 μg/ml), bafilomycin A1 (10 nM) or heparin (200 μg/ml) were added at different time points for 4 h treatment (red dotted lines in the right panel). At 48 h post-infection, HCVcc infection was analyzed by IF. Results are plotted as % of HCVcc infection compared to DMSO treated group of −1.5–2.5 h. Data shown as mean ± SD of three independent experiments in the above figures. (c) The effect of SZA on virion-cell fusion. HCV virion-cell fusion was measured by DiD fluorescence dequenching in Huh7 cells treated with SZA (20 μg/ml) in a kinetic mode. NH_4_Cl (10 mM) was introduced as a positive control. Results are plotted as relative fluorescence units after subtraction of the background (uninfected culture). Data shown as mean ± SD of triplicate wells from one independent experiment. **p < 0.01 compared to DMSO treated group. (**d**) Chemical structures of cholesterol and SZA. (**e**) Liposomes composed of 70% POPC and 30% cholesterol were incubated with Prodan (15 μM) for 15 min at room temperature, and then treated with the indicated concentrations of SZA for 30 min at 37 ^o^C. Fluorescence readings were collected as delta GPs (GP_SZA_ − GP_DMSO_) at different excitation wavelengths. Data shown as mean ± SD of three independent experiments.

We next investigated the possibility of virion-cell surface receptor-binding resulting in its delivery to the tight junction areas and subsequent internalization by clathrin-mediated endocytosis. As shown in Fig. 5a, the HCV internalization ratio compared to virus binding was not affected by SZA treatment, excluding the possible anti-HCV effect of SZA on virus internalization.

To further elucidate the entry steps targeted by SZA, we evaluated the entry kinetics of this compound. As a control, heparin markedly blocked HCVcc infection when added prior to the virus binding, but inhibited HCVcc infection less efficiently when added after the binding (Fig. 5b). However, similar to bafilomycin A1, a membrane fusion inhibitor of interfering endosomal acidification^19^, SZA had an inhibitory effect when added even 2 h after virus inoculation (Fig. 5b), indicating that SZA exerts its action late after cell entry.

To determine the effect of SZA on the fusion process, we introduced a dynamic, real-time detection assay of membrane fusion. HCVcc was labeled with a hydrophobic fluorophore DiD labeling solution, which is quenched at a high concentrations but unquenched when diluted during virion-host cell fusion, for real-time detection. Compared with the endosome acidification inhibitor NH_4_Cl, SZA more potently reduced the occurrence of membrane fusion, which indicates that these compounds are likely to display their inhibitory effects during this process (Fig. 5c). Moreover, the structure of SZA resembles cholesterol (Fig. 5d), which enables this compound to penetrate the host membrane, inhibiting viral fusion by increasing host membrane fluidity. Therefore, we evaluated the effect of SZA on lipid membrane fluidity using fluorescent dye Prodan. The liposomes composed of 70% 2-Oleoyl-1-palmitoyl-sn-glycero-3-phospho-choline (POPC) and 30% cholesterol were incubated with the indicated concentrations of SZA, and the generalized polarization (GP) of liposome membranes was assessed. Consistent with the above results of membrane fusion assay, SZA significantly increased the membrane fluidity of cholesterol-containing liposomes, as shown in the reduction of GP (Fig. 5e). Together, these results indicate that SZA inhibits HCV entry during virion-host cell fusion by increasing host membrane fluidity.

### SZA diminishes HCV cell-to-cell transmission and shows synergistic inhibitory effect with IFN-α or telaprevir

**Figure 6:**
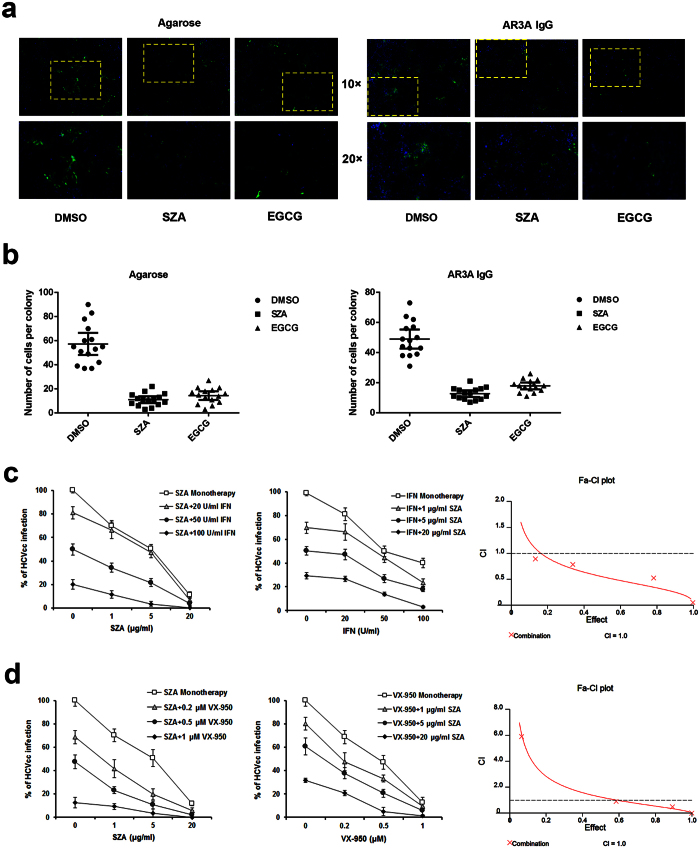
SZA blocks HCV cell-to-cell transmission and exerts synergistic effect during combination therapy. (**a**) Huh7 cells were incubated with HCVcc of JFH-1 for 2 h before overlaying with 1% agarose dissolved in culture medium or with AR3A anti-E2 neutralizing antibodies, in the presence of 20 μg/ml SZA. EGCG (50 μM) was introduced as a positive control. At 48 h post-infection, infected cells were quantified by IF. Nuclei were stained with DAPI. (**b**) Numbers of cells per positive colony were determined in 15 foci. (**c,d**) Indicated concentrations of SZA was mixed with HCVcc and incubated with Huh7 cells for 4 h. At 12 h post-infection, the indicated concentrations of IFN-α 2b or telaprevir were added in the respective cultures. Infected cells were quantified 48 h after infection by IF. Results are shown as % of HCVcc infection compared to untreated group (left and middle panels). Data shown as mean ± SD of three independent experiments. Fa-CI plots (CI versus effect) were shown in the right panels using CalcuSyn.

Followed by the infection of Huh7 cells with HCVcc, progeny viruses are propagated to the adjacent cells, resulting in the formation of local areas of infection known as foci. This mode of transmission is refractory to neutralization by agarose or anti-E2 antibodies^20^. To determine whether SZA blocks cell-to-cell transmission, the HCV-infected Huh7 cells were either overlaid with agarose-containing medium or incubated with neutralizing antibody AR3A in the presence of 20 μg/ml SZA^21^,^22^. Both methods are known to prevent reinfection of cells by newly secreted HCV particles, but allow viral cell-to-cell transmission. Three days after infection, foci were visualized by immunofluorescent (IF) assay (Fig. 6a) and the size of different foci was measured by counting the cells per focus (Fig. 6b). Epigallocatechin-3-gallate (EGCG) (50 μM) was introduced as a positive control of HCV cell-to-cell transmission^22^. A significant decrease in the cell-to-cell transmission was clearly observed in both approaches by SZA treatment, which was in accordance with the observation in EGCG treatment group (Fig. 6a,b).

Next, we investigated whether SZA could be combined with IFN-α or telaprevir. Telaprevir is an HCV NS3-4A protease inhibitor combined with IFN and RB therapy for genotype 1 patients in clinics. Our combination antiviral activity study revealed that the addition of SZA increased the efficacy of IFN or telaprevir in the inhibition of HCV infection (Fig. 6c,d, left and middle panels). In addition, the combination index (CI) of both combined therapies were above 1.0, suggesting a synergistic effect of SZA in the combination therapy (Fig. 6c,d, right panel).

### SZA impairs the establishment of HCV infection *in vivo*

**Figure 7:**
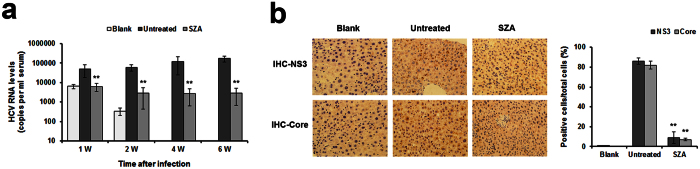
SZA diminishes HCV infection in transgenic mice. (**a**) ICR^R+^ mice were pretreated with DMSO (untreated) (n = 12) or SZA (n = 20) (5 mg per kg body weight per day) for 2 weeks before infection and 1 week after infection through intraperitoneal injection. Non-transgenic mice (n = 3) with ICR background was also injected with virus (blank). The blood samples were taken at different time points during the course of HCV infection (1/2/4/6 weeks post-infection). HCV RNA levels in different mice serum samples were tested using RT-qPCR. Data shown as mean ± SD of mice from different groups from one experiment. **p < 0.01 compared with untreated group. (**b**) IHC assay was performed on paraffin-embedded mice liver sections with anti-NS3 and anti-core antibodies. Quantification analysis was performed to calculate the positive rate of the cells. Data shown as mean ± SD of five fields under microscope from one experiment. **p < 0.01 compared with untreated group.

Finally, we assessed the ability of SZA to inhibit HCV infection *in vivo* using transgenic mice which harbored human SRB1, CD81, CLDN1 and OCLN genes (ICR^R+^ mice). A 5 mg/kg/d SZA dose was administrated via intraperitoneal injection and well tolerated in ICR^R+^ mice, as there was no significant damage to the vital organs such as the heart, liver, spleen, lungs and kidneys of the mice (Supplementary Fig. 3). The mice were then treated with SZA or DMSO (5 mg/kg/d) for 2 weeks before inoculation of JFH-1 HCVcc and 1 week after the viral challenge. SZA treatment in ICR^R+^ mice diminished HCV infection as shown by the reduction of HCV RNA levels in the serum of treated mice during the course of viral infection, while HCV RNA levels remained relatively high level in untreated mice (Fig. 7a, Supplementary Fig. 4). Furthermore, mouse liver tissue samples were subjected to HCV NS3 and core immunohistochemistry (IHC) assays. As shown in Fig. 7b, the number of positive hepatocytes in HCV NS3 or core protein of SZA-treated mice was much smaller than untreated mice, demonstrating the antiviral effect of SZA on HCV infection *in vivo*.

## Discussion

By screening schisandra fruit extracts, we identified SZA, a tetracyclic triterpene, with a genotype-independent antiviral activity against HCV. Our detailed mechanistic studies indicated that SZA prevented HCV infection by inhibiting the step after host cell surface binding and internalization of the viral particles, and by blocking intercellular spread to neighboring cells. This antiviral activity was confirmed in the PHHs, and the combination of SZA with IFN-α or telaprevir synergistically increased antiviral potency. SZA also inhibited HCV infection in ICR^R+^ mice.

HCV entry represents an attractive target for antiviral intervention, with opportunities to prevent multiple virus-receptor interactions and interfere with viral internalization or membrane fusion^23^. Several agents have been reported to inhibit HCV entry at the host cell-surface binding step^22^,^24^. Antibodies and peptides targeting the glycoproteins or cellular receptors, such as CD81, SRB1 and CLDN1, have been shown to block virus entry *in vitro* and *in vivo*^25^,^26^. The recent identified HCV entry factors, EGFR and NPC1L1, are now considered potential targets^14^,^16^. A small molecule ITX-5061, an E2 and SRB1 interaction disruptor, is entering phase II clinical trials^27^. In our view, SZA is a highly attractive antiviral drug candidate because it is active *in vitro* against all the HCV genotypes tested and innocuous.

SZA inhibits a post-binding and post-internalization step of HCV entry into target cells. It does not affect viral attachment or the lipoprotein moiety associated with the virion. Furthermore, the expression of specific cellular entry factors of target cells also remained unaltered by the treatment of SZA. Our data further suggest that SZA blocks HCV entry by inhibiting the fusion process. As the structure of SZA resembles cholesterol, a key element of HCV entry, we believe SZA exerted its action by altering the host membrane’s fluidity. However, the antiviral activity of SZA in HCV was not consistent in JEV infection, suggesting that this anti-fusion property does not involve a universal antiviral mechanism.

Schinortriterpenes are characteristic constituents of the Schisandraceae species. Among them, the antiviral effects of tetracyclic triterpenes and pentacyclic triterpenes are widely studied. They have been reported to exhibit antiviral activities against several other viruses. In the case of HIV, owing to the stereo structural similarities with cholesterol molecules, tetracyclic triterpenes showed stronger inhibition on HIV entry compared with pentacyclic triterpenes^28^. Against HCV, we assume SZA could also have stronger inhibitory effect on HCV entry into the host cell, resulting from its much closer core structure similarity with cholesterol molecules. Thus, tetracyclic triterpenes can be the lead natural products for the development of potential HCV entry inhibitors.

One of the major problems in the liver transplantation cases resulting from HCV is the reinfection of the graft, which is often observed with an accelerated progression of the liver disease^29^. Thus, the ability of SZA to inhibit HCV cell-to-cell transmission is a major asset for an entry inhibitor. Furthermore, the combination of entry, replication, and polyprotein-processing inhibitors, in the context of a multi-drug therapy, might be the effective option to reduce the emergence of resistant viruses.

In this study, we also tested the therapeutic effect of SZA *in vivo*. Although the antiviral activity could be observed in SZA-treated mice with approximately two log reduction in HCV RNA levels and obviously less positive cells of viral protein compared to the control group, HCV infection was still not eliminated completely in the infected mice. This might probably due to the unsatisfying bioavailability and metabolism of the drug. Further studies should be taken to make improvements in these fields, optimizing the *in vivo* application.

In conclusion, the schisandra-derived compound SZA has the inhibitory effects on pan-HCV genotype infections, in both cell free virus infection and cell-to-cell spread. Considering its therapeutic target differs from those of DAAs in current market, SZA has a potential as a lead compound for the development as an entry inhibitor, which should be especially used in prevention of HCV reinfection during the course of liver transplantation.

## Materials and Methods

### Chemicals

Dulbecco’s modified Eagle’s medium (DMEM), fetal bovine serum (FBS), L-glutamine, non-essential amino acids (NAAs), streptomycin and penicillin from GIBCO-Invitrogen (Carlsbad, CA), heparin, bafilomycin A1, NH_4_Cl, 2-Oleoyl-1-palmitoyl-sn-glycero-3-phospho-choline (POPC) and cholesterol from Sigma-Aldrich Corporation (St Louis, MO) and 4′,6-diamidino-2-phenylindole (DAPI) from Invitrogen (Carlsbad, CA) were used in this study. HCV NS3-4A serine protease inhibitor telaprevir, NS5A inhibitor dasatinib, HCV assembly inhibitor naringenin and (-)-epigallocatechin gallate (EGCG) were purchased from Selleck Chemicals (Houston, TX). Fluorescent dye Prodan was from Molecular Probes (Invitrogen, Carlsbad, CA). SZA and other natural compounds were extracted and isolated from the fruits of *Schisandra sphenanthera Rehd. et Wils.* as previously described^30^. The schisandra fruits were collected in Yunnan province and identified as dry fruits by professor Han-Ming Zhang. For the detailed procedures (see Supplementary materials and methods).

### Cell culture

Human hepatoma Huh7 cells and human embryonic kidney HEK 293T cells were cultured in DMEM containing 10% FBS, 1 × NAAs, 100 IU/ml of streptomycin and penicillin and 2 mM L-glutamine. Primary human hepatocytes (PHHs) (#5200) were obtained from ScienCell Research Laboratories (San Diego, CA) and cultured according to the manufacturer’s instructions^31^.

### Antibodies

Anti-CD81 monoclonal antibody (mAb) 5A6 from Santa Cruz Biotechnology (Paso Robles, CA), anti-SRB1, anti-CLDN1, anti-OCLN antibodies, Alexa 488- and horseradish peroxidase (HRP) conjugated anti-goat or anti-rabbit or anti-mouse IgG from Invitrogen (Carlsbad, CA) were used in our study. Anti-NS3 and anti-core antibodies were purchased from Abcam (Toronto, Ontario, Canada).

### Production and infection assay of cell culture derived HCV (HCVcc)

The plasmid encoding Japanese fulminant hepatitis type 1 (JFH-1) genome was provided by T. Wakita (National Institute of Infectious Diseases, Tokyo, Japan), and was used to produce HCVcc that was further concentrated and purified as previously described^22^,^32^,^33^. Briefly, the plasmid of JFH-1 was linearized to serve as a template of *in vitro* transcription to produce viral RNA using MEGAscript kit (Promega, Madison, WI). Huh7 cells were then transfected with quantified HCV RNA by electroporation. The supernatants of the cells were collected 5 days after the transfection and filtered through a 0.45 μm membrane. The HCVcc was further concentrated and purified by precipitation with 8% polyethylene glycol 6000 followed by a continuous 10–40% iodixanol gradient ultracentrifugation of the pelleted virus. The viral titer was quantified as 3 × 10^6^ focus forming units/ml. For the infection assay, Huh7 cells were seeded on a 96-well plate overnight and infected with HCVcc for 4 h at 37 ^o^C in the presence of SZA. After 48 h of infection, the cells were subjected to immunofluorescent (IF) assay for the measurement of infection.

### Production and entry assay of HCV pseudo-particles (HCVpp)

HCVpp were generated as previously described^34^,^35^. Briefly, HEK 293T cells were transfected with the plasmids encoding HCV envelope proteins, Gag/Pol, Rev and the transfer vector of pLenti6 with the green fluorescent protein gene. The HCV envelope plasmids included genotype 1a strain H77 (provided by F.L. Cosset, INSERM U758, Lyon, France), 1b strain Con1 (provided by C.M. Rice, Rockefeller University, NY, USA), genotypes 2a (clone UKN2A1.2), 4a (clone UKN4.21.16) and 5a (clone UKN5.15.7) (provided by J.K. Ball, The University of Nottingham, UK). The supernatants of the transfected cells were collected 48 h later and filtered through a 0.45 μm membrane. The vesicular stomatitis virus pseudo-particles (VSVpp) were produced as controls. During the entry assay, Huh7 were seeded on 6-well plates overnight and incubated with HCVpp for 4 h at 37 ^o^C in the presence of SZA. After 72 h of incubation, HCVpp entry into Huh7 cells was measured by flow cytometry as described^36^.

### Indirect IF assay

The infected Huh7 cells were washed, fixed with cold methanol followed by infectivity detection by examining NS5A expression with mAb 9E10^37^.

### Membrane fusion assay

The membrane fusion assay was performed according to the instructions laid down by a previous report^14^. JFH-1 HCVcc was concentrated and purified by Amicon Ultra-15 Centrifugal Filter units (Millipore). 30 μl of 1 mM Vybrant® DiD cell-labeling solution (Invitrogen, Carlsbad, CA) was then incubated with 2 × 10^6 ^FFU/ml JFH-1 HCVcc or same amount of solvent DMEM at 37 ^o^C for 30 min in the dark to generate DiD-labeled JFH-1 HCVcc (HCVcc^DiD^) or media control. HCVcc^DiD^ was then purified by iodixanol gradient ultracentrifugation as previously described^38^. Huh7 cells were seeded the previous day and incubated with the purified HCVcc^DiD^ or mock culture in the presence of DMSO, SZA or NH_4_Cl in a 96-well plate which was subjected to microplate reader for DiD dequenching measurement every 15 min over the course of 8 h at 37 ^o^C, at 640 nm (excitation) and 670 nm (emission) for a total of 32 cycles in a kinetic mode. The background relative fluorescence units (RFU) values of DiD in the wells free of cells and mock-infected wells with media control were subtracted from the original RFU values in the experiment wells.

### Membrane fluidity assay

The fluorescent dye Prodan was used to evaluate the effect of SZA on membrane fluidity. 200 μM liposomes composed of 70% POPC and 30% cholesterol were incubated with Prodan (15 μM) for 15 min at room temperature in the dark, and then treated with the indicated concentrations of SZA for 30 min at 37 ^o^C. The mixture was subsequently transferred into a white 384-well plate for fluorescence detection using the TECAN Infinite 200 PRO microplate reader with excitation wavelength from 310 to 350 nm and emission spectra recorded at 440 and 480 nm. GP was calculated according to a previous study^39^. The background signal with pure liposomes was subtracted from the original data. Fluorescence readings were collected as delta GPs (GP_SZA_ − GP_DMSO_) at different excitation wavelengths.

### Cell-to-cell transmission assay

The HCV cell-to-cell transmission assay was performed in two ways^22^. Briefly, Huh7 cells were infected with the purified HCVcc of JFH-1 for 2 h. The virus was then removed and the cells were overlaid with culture medium containing 1% agarose or AR3A anti-E2 neutralizing antibodies in the presence of 20 μg/ml SZA^21^. At 48 h post-infection, the infectivity was quantified by performing IF, and nuclei of the cells were stained with DAPI. The cell number was determined in 15 colonies.

### Animals and relevant infection assays

Transgenic ICR mice which resembled transgenic mice expressing human CD81 and OCLN genes (C/O^Tg^) harboring human SRB1, CD81, CLDN1 and OCLN genes (ICR^R+^) were kindly provided by Prof. Xiao-Lian Zhang and used in the study (not published)^40^. All mouse experiments were performed according to the guide for the care and use of medical laboratory animals, and approved by the Animal Care and Use Committee at the Second Military Medical University. 8 week-old male mice were divided into two groups and injected with JFH-1 HCVcc of 1.0 × 10^6^ genome copies. A non-transgenic mouse with ICR background was also injected with the virus to serve as a blank control. Treatment of SZA or DMSO (5 mg/kg) was given via intraperitoneal injection every day for 2 weeks before infection and 1 week after infection. Blood samples were taken using retro-orbital puncture after virus infection. Total RNA of the serum was extracted using TRIzol LS reagent (Invitrogen, Carlsbad, CA), and HCV RNA level was detected by HCV RNA-PCR-Fluorescence Quantitative Diagnostic Kit (Kehua Bio-engineering Corporation, Shanghai, China). Immunohistochemistry (IHC) assay was performed on paraffin-embedded mice liver sections with anti-NS3 and anti-core antibodies.

### Statistical analysis

The bar and curve graphs, showing mean and standard deviation of at least three independent experiments, were plotted. Statistical analyses were performed using SPSS 17.0. A p value of <0.05 in the Student’s t-test was considered as statistically significant.

Additional experimental procedures are listed in the Supplementary materials and methods.

## Additional Information

**How to cite this article**: Qian, X.-J. *et al.* A Schisandra-Derived Compound Schizandronic Acid Inhibits Entry of Pan-HCV Genotypes into Human Hepatocytes. *Sci. Rep.* **6**, 27268; doi: 10.1038/srep27268 (2016).

## Supplementary Material

Supplementary Information

## References

[b1] Thomas D. L. Global control of hepatitis C: where challenge meets opportunity. Nature medicine 19, 850–858, doi: 10.1038/nm.3184 (2013). PMC4937625 23836235

[b2] Manns M. P., Wedemeyer H. & Cornberg M. Treating viral hepatitis C: efficacy, side effects, and complications. Gut 55, 1350–1359, doi: 10.1136/gut.2005.076646 (2006). 16905701 PMC1860034

[b3] Kohli A., Shaffer A., Sherman A. & Kottilil S. Treatment of hepatitis C: a systematic review. Jama 312, 631–640, doi: 10.1001/jama.2014.7085 (2014). 25117132

[b4] Cox A. L. MEDICINE. Global control of hepatitis C virus. Science 349, 790–791, doi: 10.1126/science.aad1302 (2015). 26293940

[b5] Pelosi L. A., Voss S., Liu M., Gao M. & Lemm J. A. Effect on hepatitis C virus replication of combinations of direct-acting antivirals, including NS5A inhibitor daclatasvir. Antimicrobial agents and chemotherapy 56, 5230–5239, doi: 10.1128/AAC.01209-12 (2012). 22850513 PMC3457360

[b6] Gritsenko D. & Hughes G. Ledipasvir/Sofosbuvir (Harvoni): improving options for hepatitis C virus infection. P & T: a peer-reviewed journal for formulary management 40, 256–276 (2015). 25859119 PMC4378517

[b7] A 4-drug combination (Viekira Pak) for hepatitis C. Jama 313, 1857–1858, doi: 10.1001/jama.2015.4562 (2015). 25965236

[b8] Lam B. P., Jeffers T., Younoszai Z., Fazel Y. & Younossi Z. M. The changing landscape of hepatitis C virus therapy: focus on interferon-free treatment. Therapeutic advances in gastroenterology 8, 298–312, doi: 10.1177/1756283X15587481 (2015). 26327920 PMC4530432

[b9] Wyles D. L. Antiviral resistance and the future landscape of hepatitis C virus infection therapy. J Infect Dis 207 Suppl 1, S33–39, doi: 10.1093/infdis/jis761 (2013). 23390303

[b10] Syed G. H. *et al.* Hepatitis C virus stimulates low-density lipoprotein receptor expression to facilitate viral propagation. J Virol 88, 2519–2529, doi: 10.1128/JVI.02727-13 (2014). 24352472 PMC3958050

[b11] Barth H. *et al.* Cellular binding of hepatitis C virus envelope glycoprotein E2 requires cell surface heparan sulfate. J Biol Chem 278, 41003–41012, doi: 10.1074/jbc.M302267200 (2003). 12867431

[b12] Douam F., Lavillette D. & Cosset F. L. The mechanism of HCV entry into host cells. Prog Mol Biol Transl Sci 129, 63–107, doi: 10.1016/bs.pmbts.2014.10.003 (2015). 25595801

[b13] Blanchard E. *et al.* Hepatitis C virus entry depends on clathrin-mediated endocytosis. J Virol 80, 6964–6972, doi: 10.1128/JVI.00024-06 (2006). 16809302 PMC1489042

[b14] Sainz B. Jr. *et al.* Identification of the Niemann-Pick C1-like 1 cholesterol absorption receptor as a new hepatitis C virus entry factor. Nat Med 18, 281–285, doi: 10.1038/nm.2581 (2012). 22231557 PMC3530957

[b15] Martin D. N. & Uprichard S. L. Identification of transferrin receptor 1 as a hepatitis C virus entry factor. Proc Natl Acad Sci USA 110, 10777–10782, doi: 10.1073/pnas.1301764110 (2013). 23754414 PMC3696786

[b16] Lupberger J. *et al.* EGFR and EphA2 are host factors for hepatitis C virus entry and possible targets for antiviral therapy. Nature medicine 17, 589–595, doi: 10.1038/nm.2341 (2011). PMC3938446 21516087

[b17] Azzam H. S., Goertz C., Fritts M. & Jonas W. B. Natural products and chronic hepatitis C virus. Liver Int 27, 17–25, doi: 10.1111/j.1478-3231.2006.01408.x (2007). 17241377

[b18] Melhem A. *et al.* Treatment of chronic hepatitis C virus infection via antioxidants: results of a phase I clinical trial. J Clin Gastroenterol 39, 737–742 (2005). 16082287 10.1097/01.mcg.0000174023.73472.29

[b19] Meertens L., Bertaux C. & Dragic T. Hepatitis C virus entry requires a critical postinternalization step and delivery to early endosomes via clathrin-coated vesicles. Journal of virology 80, 11571–11578, doi: 10.1128/JVI.01717-06 (2006). 17005647 PMC1642584

[b20] Brimacombe C. L. *et al.* Neutralizing antibody-resistant hepatitis C virus cell-to-cell transmission. Journal of virology 85, 596–605, doi: 10.1128/JVI.01592-10 (2011). 20962076 PMC3014195

[b21] Law M. *et al.* Broadly neutralizing antibodies protect against hepatitis C virus quasispecies challenge. Nature medicine 14, 25–27, doi: 10.1038/nm1698 (2008). 18064037

[b22] Calland N. *et al.* (-)-Epigallocatechin-3-gallate is a new inhibitor of hepatitis C virus entry. Hepatology 55, 720–729, doi: 10.1002/hep.24803 (2012). 22105803

[b23] Zhu Y. Z., Qian X. J., Zhao P. & Qi Z. T. How hepatitis C virus invades hepatocytes: the mystery of viral entry. World J Gastroenterol 20, 3457–3467, doi: 10.3748/wjg.v20.i13.3457 (2014). 24707128 PMC3974512

[b24] Ciesek S. *et al.* The green tea polyphenol, epigallocatechin-3-gallate, inhibits hepatitis C virus entry. Hepatology 54, 1947–1955, doi: 10.1002/hep.24610 (2011). 21837753

[b25] Krieger S. E. *et al.* Inhibition of hepatitis C virus infection by anti-claudin-1 antibodies is mediated by neutralization of E2-CD81-claudin-1 associations. Hepatology 51, 1144–1157, doi: 10.1002/hep.23445 (2010). 20069648

[b26] Si Y. *et al.* A human claudin-1-derived peptide inhibits hepatitis C virus entry. Hepatology 56, 507–515, doi: 10.1002/hep.25685 (2012). 22378192 PMC3406249

[b27] Syder A. J. *et al.* Small molecule scavenger receptor BI antagonists are potent HCV entry inhibitors. J Hepatol 54, 48–55, doi: 10.1016/j.jhep.2010.06.024 (2011). 20932595

[b28] Xu L. J., Peng Z. G., Chen H. S., Wang J. & Xiao P. G. Bioactive triterpenoids from Kadsura heteroclita. Chem Biodivers 7, 2289–2295, doi: 10.1002/cbdv.200900173 (2010). 20860030

[b29] Brown R. S. & Hepatitis C. and liver transplantation. Nature 436, 973–978, doi: 10.1038/nature04083 (2005). 16107838

[b30] Du J. L. *et al.* Chemical and Biologically Active Constituents of Schisandra sphenanthera Rehd. et. Asian Journal of Chemistry 25, 2321–2322 (2013).

[b31] Nahmias Y., Casali M., Barbe L., Berthiaume F. & Yarmush M. L. Liver endothelial cells promote LDL-R expression and the uptake of HCV-like particles in primary rat and human hepatocytes. Hepatology 43, 257–265, doi: 10.1002/hep.21016 (2006). 16440337

[b32] Goueslain L. *et al.* Identification of GBF1 as a cellular factor required for hepatitis C virus RNA replication. J Virol 84, 773–787, doi: 10.1128/JVI.01190-09 (2010). 19906930 PMC2798365

[b33] Wakita T. *et al.* Production of infectious hepatitis C virus in tissue culture from a cloned viral genome. Nature medicine 11, 791–796, doi: 10.1038/nm1268 (2005). PMC2918402 15951748

[b34] Guan M. *et al.* Three different functional microdomains in the hepatitis C virus hypervariable region 1 (HVR1) mediate entry and immune evasion. J Biol Chem 287, 35631–35645, doi: 10.1074/jbc.M112.382341 (2012). 22927442 PMC3471721

[b35] Tong Y. *et al.* Tupaia CD81, SR-BI, claudin-1, and occludin support hepatitis C virus infection. Journal of virology 85, 2793–2802, doi: 10.1128/JVI.01818-10 (2011). 21177818 PMC3067968

[b36] Bartosch B., Dubuisson J. & Cosset F. L. Infectious hepatitis C virus pseudo-particles containing functional E1-E2 envelope protein complexes. The Journal of experimental medicine 197, 633–642 (2003). 12615904 10.1084/jem.20021756 PMC2193821

[b37] Chen Y. *et al.* Interferon-inducible cholesterol-25-hydroxylase inhibits hepatitis C virus replication via distinct mechanisms. Scientific reports 4, 7242, doi: 10.1038/srep07242 (2014). 25467815 PMC4252895

[b38] Sabahi A. *et al.* The rate of hepatitis C virus infection initiation *in vitro* is directly related to particle density. Virology 407, 110–119, doi: 10.1016/j.virol.2010.07.026 (2010). 20800257 PMC2946418

[b39] Chamoun-Emanuelli A. M. *et al.* Phenothiazines inhibit hepatitis C virus entry, likely by increasing the fluidity of cholesterol-rich membranes. Antimicrobial agents and chemotherapy 57, 2571–2581, doi: 10.1128/AAC.02593-12 (2013). 23529728 PMC3716126

[b40] Chen J. *et al.* Persistent hepatitis C virus infections and hepatopathological manifestations in immune-competent humanized mice. Cell research 24, 1050–1066, doi: 10.1038/cr.2014.116 (2014). 25155355 PMC4152738

