# Supplementary material for: A Schisandra-Derived Compound Schizandronic Acid Inhibits Entry of Pan-HCV Genotypes into Human Hepatocytes
Source: Sci Rep. 2016 Jun 2;6:27268. doi: 10.1038/srep27268 (PMC4890123; doi:10.1038/srep27268)
Supplement: Supplementary Information [file srep27268-s1.doc]

**Supplementary information to:**

**A Schisandra-Derived Compound Schizandronic Acid Inhibits Entry of Pan-HCV Genotypes into Human Hepatocytes**

**Authors:** Xi-Jing Qian^1,+^, Xiao-Lian Zhang^2,+^, Ping Zhao^1^, Yong-Sheng Jin^3^, Hai-Sheng Chen^4^, Qing-Qiang Xu^1^, Hao Ren^1^, Shi-Ying Zhu^1^, Hai-Lin Tang^1^, Yong-Zhe Zhu^1^,*, Zhong-Tian Qi^1^,*

**Affiliations:** ^1^Department of Microbiology, Shanghai Key Laboratory of Medical Biodefense, Second Military Medical University, Shanghai 200433, China

^2^State Key Laboratory of Virology, Medical Research Institute of Wuhan University and Department of Immunology and Hubei Province Key Laboratory of Allergy and Immunology, Wuhan University School of Medicine, 185 Donghu Road, Wuhan 430071, Hubei Province, China

^3^Department of Organic Chemistry, College of Pharmacy, Second Military Medical University, Shanghai 200433, China

^4^Department of Phytochemistry, College of Pharmacy, Second Military Medical University, Shanghai 200433, China

^+^These authors contributed equally to this work

*Corresponding authors

**Contact Information of Corresponding Authors:**

Zhong-Tian Qi, Ph.D., M.D., Department of Microbiology, Shanghai Key Laboratory of Medical Biodefense, Second Military Medical University, 800, Xiangyin Road, Shanghai 200433, China. Tel/Fax: (86)21-81870988. E-mail: qizt@smmu.edu.cn.

Yong-Zhe Zhu, Ph.D., Department of Microbiology, Shanghai Key Laboratory of Medical Biodefense, Second Military Medical University, 800, Xiangyin Road, Shanghai 200433, China. Tel/Fax: (86)21-81870990. E-mail: zhuyongzhe1984@sina.com.

**Supplementary Materials and Methods**

***Extraction and purification of the compounds.*** The plant material was collected in Yunnan province and identified as dry fruit of *schisandra sphenanthera Rehd. et Wils* by professor Han-Ming Zhang (College of Pharmacy, Second Military Medical University). A voucher specimen (20011010) is deposited in the herbarium of the college. The ripened, air-dried, and powdered fruits were used to prepare the extract with 80% EtOH. The extracted material was vacuum dried to obtain a residue, which was well-distributed in distilled water and then extracted with petroleum ether, EtOAc, and water-saturated n-BuOH successively to give petroleum ether partition, EtOAc partition and n-BuOH partition. EtOAc partition was subjected to silica gel CC (petroleum ether- EtOAc and CHCl_3_-MeOH), Sephadex LH-20 gel CC (CHCl_3_-MeOH, MeOH, MeOH-H_2_O) and RP18 (MeOH-H_2_O) repeatedly to yield monomers, whose structures were elucidated by mass spectrometry (MS) and nuclear magnetic resonance (NMR) analysis (Supplementary figure 1), and compared with authentic sample The relative content of SZA in *schisandra sphenanthera* was determined by Shimadzu LC-2010 high performance liquid chromatography (HPLC) system (mobile phase: MeOH: 0.2% phosphoric acid solution=95:5; flow rate:1.0 ml/min; Dikma 5 μm 250 mm×4.6 mm C-18 column, T=30 °C; detection wavelength: 209 nm). The yield of SZA obtained per 100 g of the fruit was 10.18 mg. The purity of SZA was 95.6%, and it was dissolved at a final concentration less than 1% DMSO for further experiments.

***Physical and spectral properties of SZA.*** White solid (chloroform), C_30_H_46_O_3_, (CDCl_3_), mp:162~164 °C; Bromocresol green test: positive; IR: 1712cm^-1^ (C=O), 1680, 1637 cm^-1^ (C=C, COOH); EI-MS m/z: 454 [M]^+^ (65), 439(39), 421(14), 355(30), 342(15), 313(50), 235(30), 175(42), 95(100).

^1^H-NMR(δ, CDCl_3_, 600 MHz ) : 0.55(1H, d, *J*=4.3 Hz，H-19a ), 0.76(1H, d, *J*=4.3 Hz, H-19b), 6.07(1H, t, *J*=7.6 Hz, H-24), 0.89(3H, s, Me-18), 0.87(3H, d, *J*=3.8 Hz, Me-21), 1.90(3H, s, Me-27), 1.02(3H, s, Me-28), 1.08(3H, s, Me-29), 0.97(3H, s, Me-30).

^13^C-NMR(δ, CDCl_3_, 150 MHz): 35.5(C-1), 37.5(C-2), 216.7(C-3), 50.2(C-4), 48.4(C-5), 21.5(C-6), 28.1(C-7), 47.9(C-8), 21.1(C-9), 25.9(C-10), 26.7(C-11), 35.8(C-12), 45.3(C-13), 48.7(C-14), 33.4(C-15), 26.9(C-16), 52.2(C-17), 18.1(C-18), 29.6(C-19), 36.0(C-20), 18.1(C-21), 32.7(C-22), 25.9(C-23), 147.4(C-24), 125.8(C-25), 173.4(C-26), 20.5(C-27), 19.3(C-28), 22.2(C-29), 20.5(C-30).

***Japanese Encephalitis Virus infection assay.*** JEV strain SA14 was a gift from Yong-Xin Yu (National Institute for the Control of Pharmaceutical and Biological Products, Beijing, China), and propagated in BHK-21 cells. The virus was concentrated and purified by a discontinuous sucrose gradient ultracentrifugation as described previously [^1^](#_ENREF_1). The Huh7 cells were infected with 0.1 MOI of JEV in the presence of SZA for 1 h at 37 ^o^C. At 36 h post-infection, the cells were fixed with 4% paraformaldehyde for 20 min at room temperature followed by 10 min permeabilization with 0.1% Triton X-100. The infectivity was detected by examining JEV E protein with anti-JEV E mouse mAb (a gift from the Fourth Military Medical University, Xi’an, China).

***Viability assay.*** The Huh7 cells or PHHs were incubated with the compounds for 24 h before the viability was detected using Cell Counting Kit-8 (CCK8) (Beyotime Biotechnology, China).

***Western Blotting and Flow Cytometry.*** Western blotting and flow cytometry were performed to analyze the cell surface HCV receptor expression level as previously described [^2^](#_ENREF_2). Briefly, the overnight seeded Huh7 cells were incubated with SZA for 4 h, and then lysed to detect the expression levels of HCV entry factors SRB1, CLDN1 and OCLN by western-blotting. CD81 expression level was determined by flow cytometry.

***Freeze and Thaw Lysis of HCV-transfected cells.*** The intracellular virus infectivity of the HCV-electroporated cells was detected by freeze and thaw lysis assay as previously described [^3^](#_ENREF_3). Briefly, the Huh7 cells were transfected with HCV RNA of JFH-1, and then seeded on 6-well plates for 4 h. The cells were then treated with SZA for 24 h before the cell supernatants were collected 48 h after transfection to determine extracellular viral infectivity. The cells were then subjected to three cycles of freeze and thaw in liquid nitrogen and a 37 ^o^C incubator to measure the intracellular viral infectivity by infecting naïve Huh7 cells.

***Iodixanol density-gradient fractionation.*** SZA was incubated with concentrated and purified HCVcc of JFH-1 at 37 ^o^C for 4 h before the compound was removed by Amicon Ultra-15 Centrifugal Filter units (Millipore). The virus was then loaded on a continuous iodixanol gradients (10-40%) for 16 h ultracentrifugation at 146,000 g at 4 ^o^C. 1 ml of each fraction (total 10 fractions) was collected and weighed to calculate the density. HCV RNA level was quantified by RT-qPCR, and HCV infectivity of each fraction was determined by reinfection of naïve Huh7 cells.

***HCVcc Binding and Internalization Assay.*** The Huh7 cells were infected with concentrated and purified HCVcc in a 24-well plate for 90 minutes at 4 ^o^C in the presence of SZA. The cells were then washed three times with cold phosphate buffered saline (PBS), and total RNA was extracted with TRIzol reagent (Invitrogen). HCV RNA levels were quantified by RT-qPCR. Internalization assay was measured as described [^4^](#_ENREF_4). Briefly, the virus was incubated with the Huh7 cells in a 24-well plate at 4 ^o^C for 90 minutes to concentrate on the cell surface. The plate was then put in a 37 ^o^C incubator for 30 minutes to allow virus internalization before the cells were treated with trypsinization at 4 ^o^C to remove the non-internalized virions. The internalized viral particles were determined by RT-qPCR.

**Supplementary table**

**The name and structure of numbered schisandra-derived compounds**

| **Schisandra-derived compounds** | | |
| --- | --- | --- |
| **No** | **Name** | **Structure** |
|
| **SY-01** | Nonadecanoic acid | 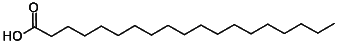 |
| **SY-02** | Cycloartenol |  |
| **SY-03** | Seco-coccinic acid A |  |
| **SY-04** | Seco-coccinic acid E |  |
| **SY-05** | Coccinelignan C |  |
| **SY-08** | Caffeic acid |  |
| **SY-10** | 3-n-butoxypolyethylene -4- methoxycinnamic acid |  |
| **SY-11** | 3-methoxybenzaldehyde |  |
| **SY-12** | Coccinelignan A |  |
| **SY-13** | Coccinelignan B |  |
| **SY-14** | Ferulic acid |  |
| **SY-15** | 2,4-Dihydroxybenzoic acid |  |
| **SY-16** | 3,​7-​dimethyl-1,​6-​Octadien-​3-​ol |  |
| **SY-17** | Linalool |  |
| **SY-19** | Lariciresnol |  |
| **SY-22** | Vinillin |  |
| **SY-23** | Sitosterol |  |
| **SY-25** | Frambinone |  |
| **SY-26** | p-hydroxybenzaldehyde |  |
| **SY-27** | ω-hydroxypropioguaiacone |  |
| **SY-31** | 4,4'-±±2R,3R-2,3-dimethylbutane-1,4-diylbis±2-methoxyphenol |  |
| **SY-33** | 3-hydroxy-12-acetoxylcoccinic acid |  |
| **SY-50** | Schisantherins P |  |
| **SY-60** | 3-hydroxy-12-hydroxylcoccinic acid |  |
| **SY-64** | Stigmasterol |  |
| **SY-72** | Nigranic acid |  |
| **SY-73** | Schizandronic acid | 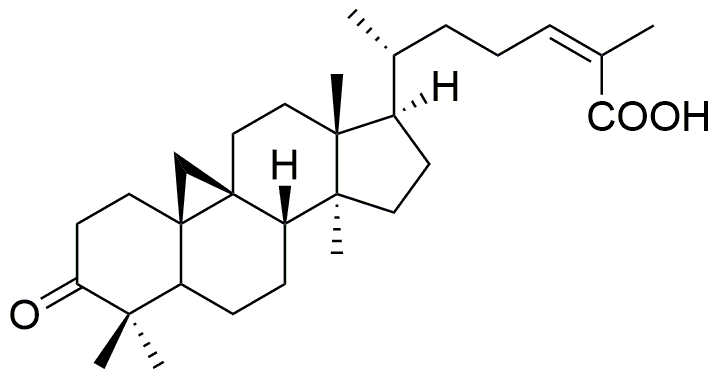 |
| **SY-74** | Pistagremic acid |  |
| **SY-75** | Neokadsuranic acid A |  |
| **SY-76** | Schisanhenol |  |
| **SY-77** | Schisandrone |  |

**Supplementary figure 1**

**
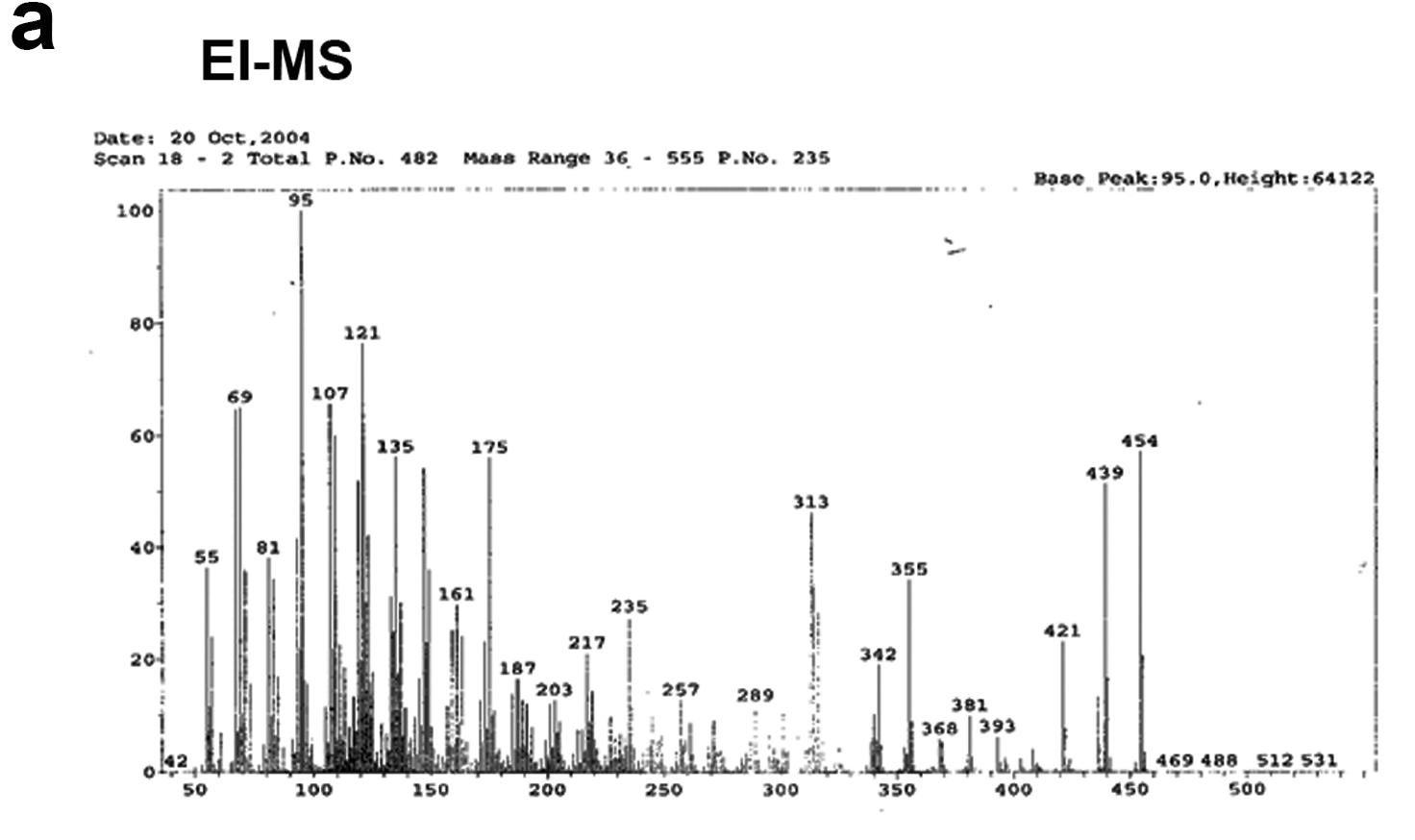
**

**
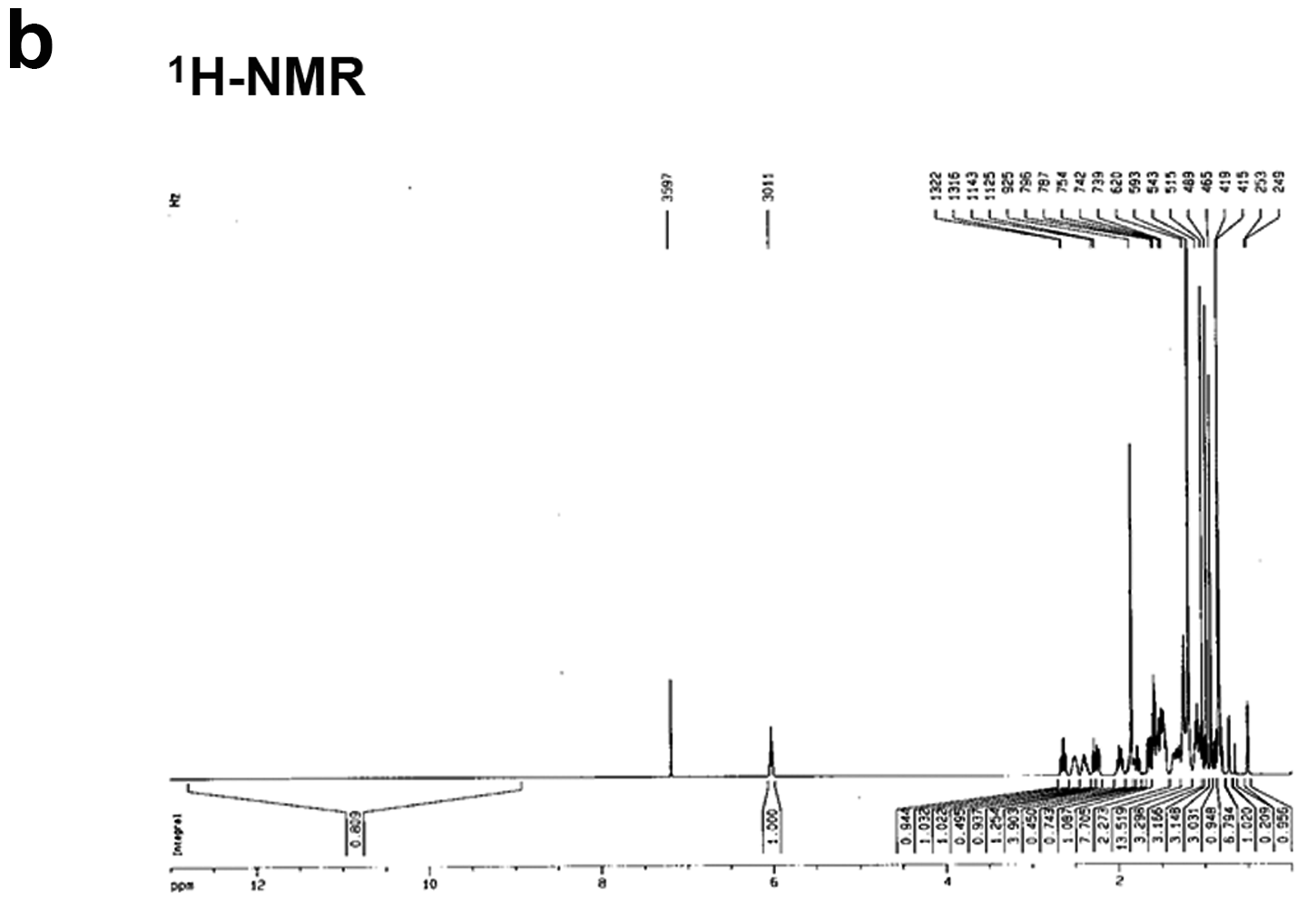
**

**
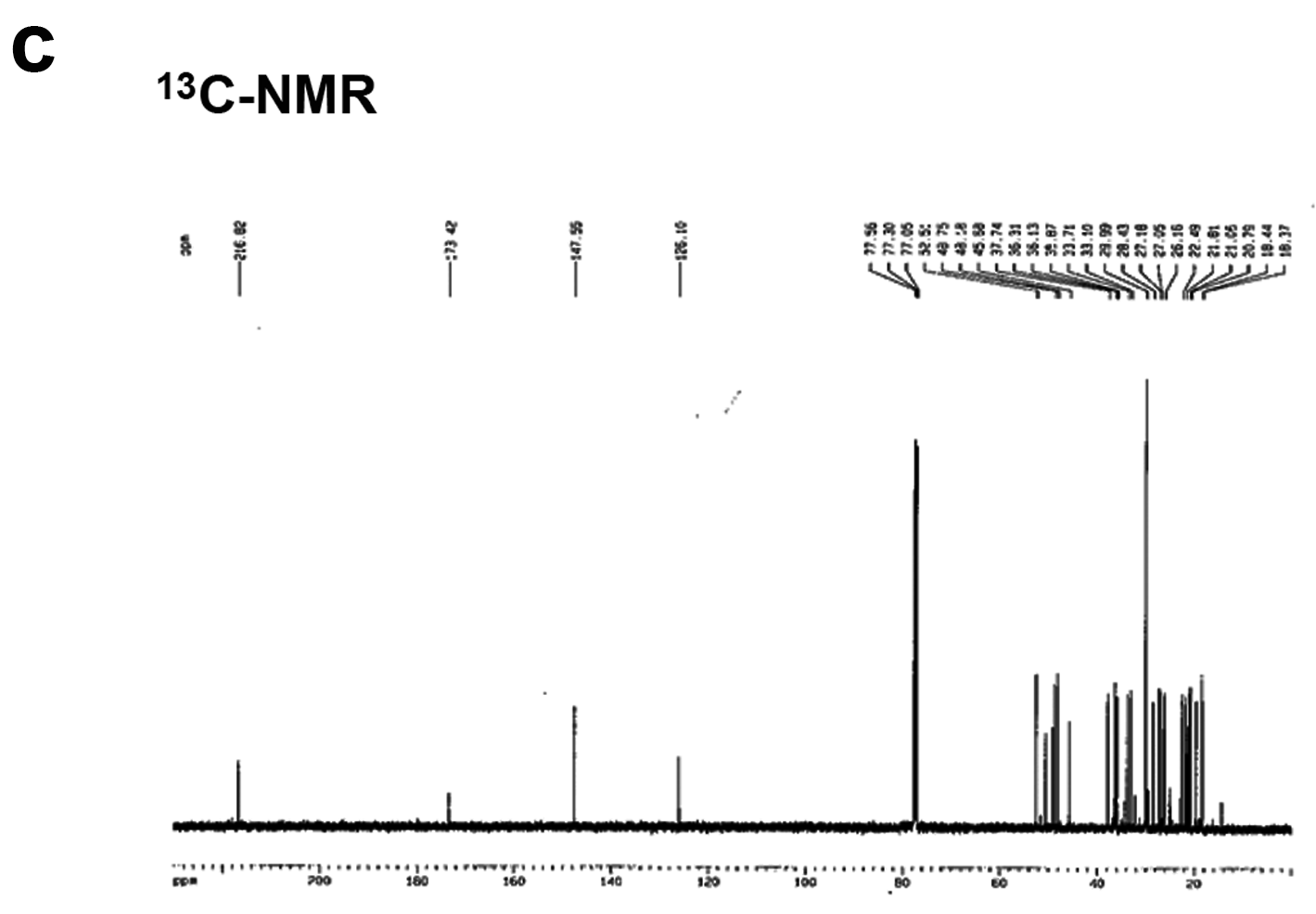
**

**
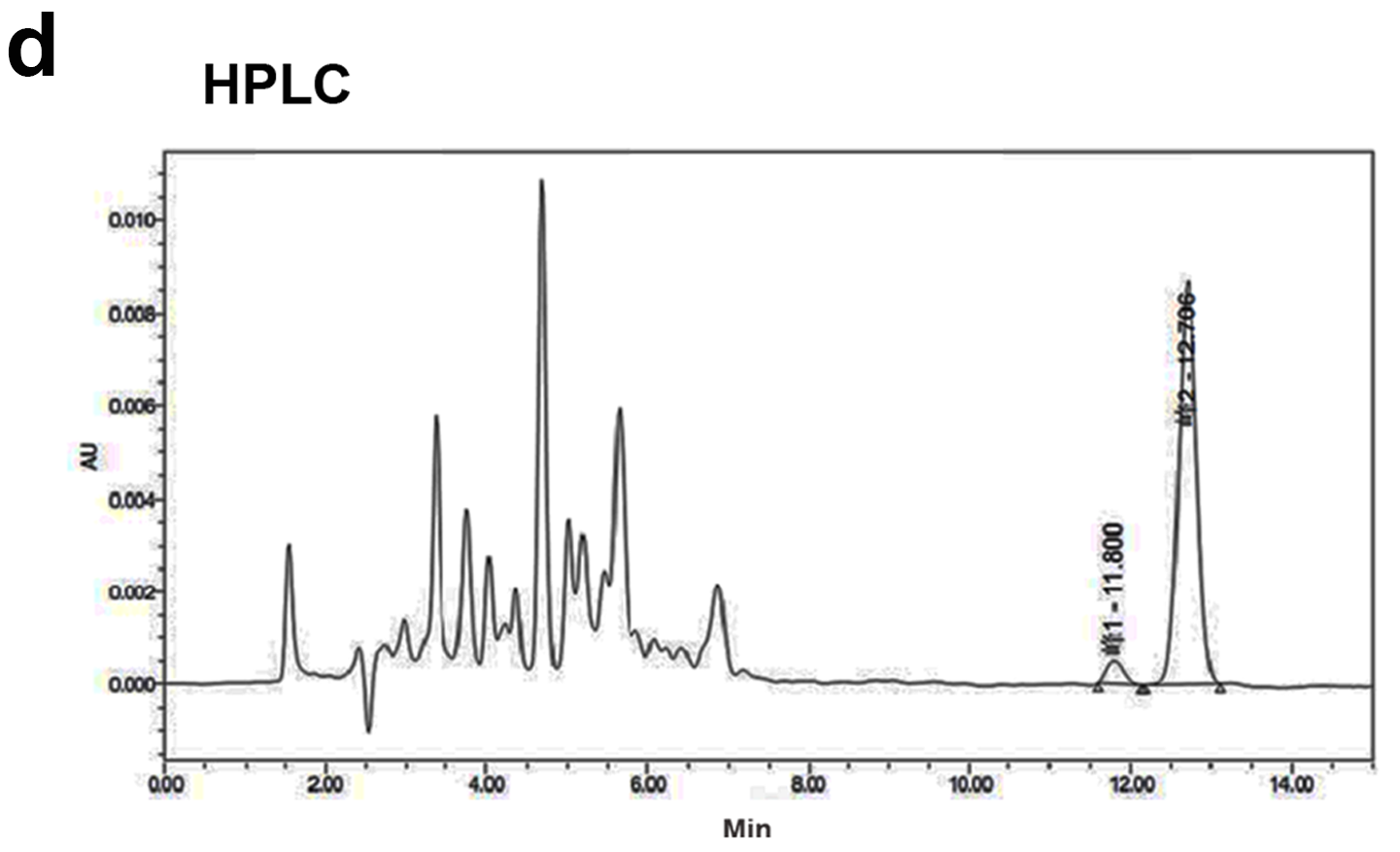
**

**Supplementary Figure 1.** Chemical identification analysis of SZA. (a) Electron impact mass spectrometry (EI-MS) analysis of SZA. (b) ^1^H-NMR of SZA (CDCl_3_, 600 MHz). (c) ^13^C-NMR of SZA (CDCl_3_, 150 MHz). (d) HPLC analysis of SZA.

**Supplementary figure 2**

**
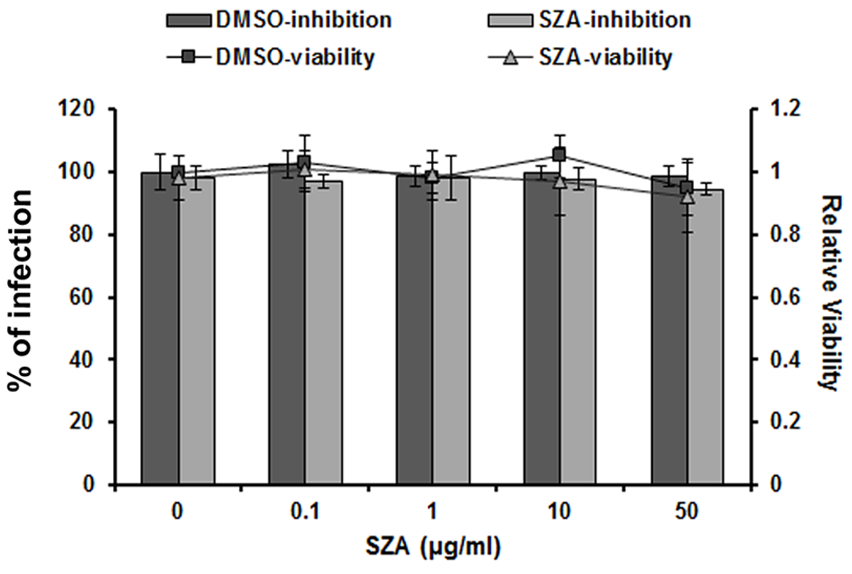
**

**Supplementary Figure 2.** Anti-JEV activity of SZA. Huh7 cells were infected with JEV (MOI=0.1) in the presence of indicated concentrations of SZA for 1 h at 37 ^o^C. At 36 h post-infection, the infectivity of JEV was detected by examining JEV E protein with anti-JEV E mouse mAb. Results were plotted as % of infection compared to the DMSO treated group in parallel.

**Supplementary figure 3**

**
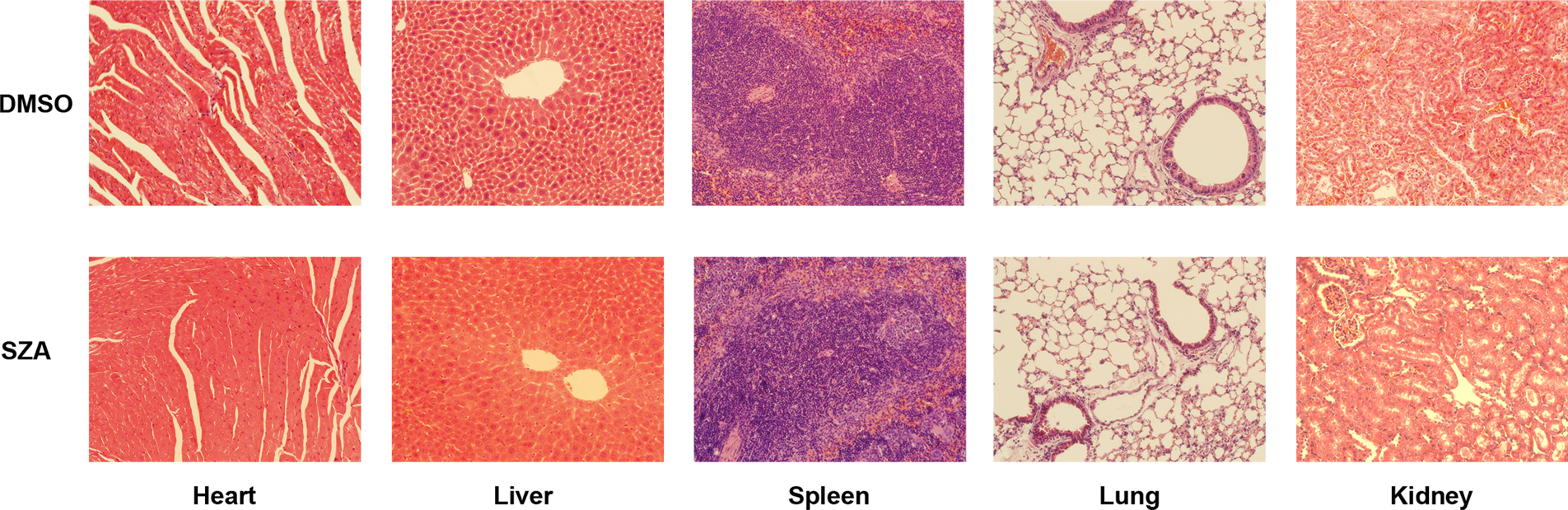
**

**Supplementary Figure 3.** Hematoxylin and eosin (H&E) stain of relevant organs (heart, liver, spleen, lung and kidney) of ICR^R+^ mice.

**Supplementary figure 4**

**
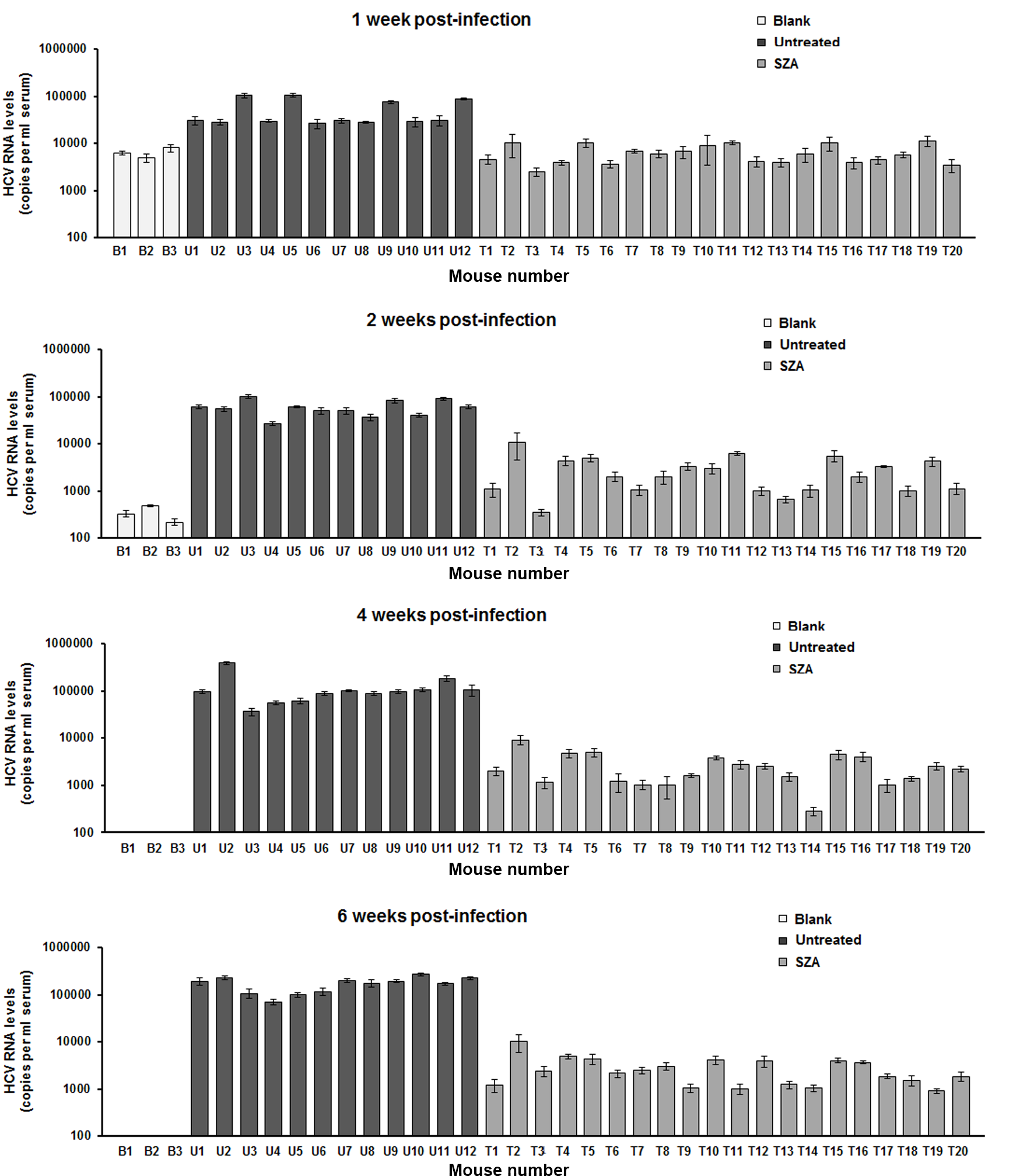
**

**Supplementary Figure 4.** ICR^R+^ mice were pretreated with DMSO (U1, U2…) (n=12) or SZA (T1, T2…) (n=20) (5 mg per kg body weight per day) for 2 weeks before infection and 1 week after infection through intraperitoneal injection. Non-transgenic mice (n=3) with ICR background was also injected with virus (B1, B2…). Serum HCV RNA levels were tested at different time points post-infection using RT-qPCR. Data were shown as mean ± SD from triplicate wells of 1 independent experiment.

**References**

1 Das, S., Laxminarayana, S. V., Chandra, N., Ravi, V. & Desai, A. Heat shock protein 70 on Neuro2a cells is a putative receptor for Japanese encephalitis virus. *Virology* **385**, 47-57, doi:10.1016/j.virol.2008.10.025 (2009).

2 Zhu, Y. Z. *et al.* Significance of palmitoylation of CD81 on its association with tetraspanin-enriched microdomains and mediating hepatitis C virus cell entry. *Virology* **429**, 112-123, doi:10.1016/j.virol.2012.03.002 (2012).

3 Haid, S. *et al.* A plant-derived flavonoid inhibits entry of all HCV genotypes into human hepatocytes. *Gastroenterology* **143**, 213-222 e215, doi:10.1053/j.gastro.2012.03.036 (2012).

4 Vausselin, T. *et al.* The antimalarial ferroquine is an inhibitor of hepatitis C virus. *Hepatology* **58**, 86-97, doi:10.1002/hep.26273 (2013).
